# Synthesis of a Dehydroabietyl Derivative Bearing a 2-(2′-Hydroxyphenyl)Benzimidazole Unit and Its Selective Cu^2+^ Chemosensing

**DOI:** 10.3390/molecules16010100

**Published:** 2010-12-28

**Authors:** Ying-Chun Wang, Lu-Zhi Liu, Ying-Ming Pan, Heng-Shan Wang

**Affiliations:** 1 College of Chemistry and Chemical Engineering, Jishou University, Jishou 416000, Hunan Province, China; E-Mail: wang_yingchun2010@yahoo.com.cn (Y-C.W.); 2 Key Laboratory of Medicinal Chemical Resources and Molecular Engineering, College of Chemistry and Chemical Enginesering, Guang xi Normal University, Guilin 541004, Guangxi Province, China; E-Mails: liuluzhi2010@hotmail.com (L.-Z.L.); panym2004@yahoo.com.cn (Y.-M.P.)

**Keywords:** dehydroabietic acid, absorption, fluorescence, copper, metal-ion recognition

## Abstract

A dehydroabietyl derivative **2** bearing a 2-(2′-hydroxyphenyl)benzimidazole unit was synthesized and its sensing behaviors toward metal ions were investigated by UV-Vis and fluorescence spectroscopy methods. In THF solution, compound **2** exhibited excellent selectivity for Cu(II) over miscellaneous other metal ions including Cr(II), Mn(II), Co(II), Ni(II), Zn(II), Cd(II), Al(III), Mg(II), Pb(II), Hg(II), Na(I), Li(I) and K(I) evidenced through the quenching of the fluorescence of the benzimidazole fragment. The reaction between **2** and Cu^2+^ was found to be stoichiometric with the formation of a 1:1 complex.

## 1. Introduction 

Recently, the design and synthesis of fluorescent organic compounds for chemical sensors has been an active research area because of their possible applications in the environmental and bio-analytical fields [1,2,3,4]. 2-(2′-Hydroxyphenyl)benzimidazole and its derivatives are attractive fluorescent compound candidates that display large Stokes shifts and high fluorescence quantum yields, and present great photophysical stability ascribed to an excited state intramolecular proton transfer (ESIPT) mechanism [5,6]. They have been studied as laser dyes [7], polymer UV-light stabilizers [8] and fluorescent probes for labelling proteins [9]. Moreover, they are excellent photoluminescent materials. In recent years, more attention has been paid to their complexes and those of other similar *N,O*-donor ligands with metal ions and non-metal ions due to their potential role as electroluminescent materials in organic light emitting diodes [10,11]. Nevertheless, there have been few reports on the use of the2-(2′-hydroxyphenyl)benzimidazole unit as a fluorophore and recognition site for metal-ion recognition in organic media. Here we report the design and synthesis of a dehydroabietyl derivative **2** bearing a 2-(2′-hydroxyphenyl)benzimidazole unit that shows high selectivity towards Cu^2+^ by the quenching of the fluorescence of the benzimidazole fragment. The influence of different metal cations on the fluorescence intensity of compound **2** in THF is also discussed.

## 2. Results and Discussion

**Scheme 1:**
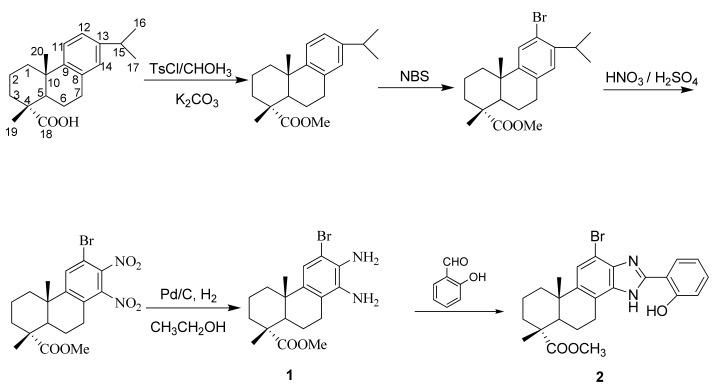
Outline of the synthesis route for compound **2**.

Compound **2** was synthesized as outlined in Scheme 1. The starting material, dehydroabietic acid, was obtained from commercially disproportionated rosin and purified by repeated crystallisation of the corresponding ethanolamine salt [12]. The intermediate 12-bromo-13,14-diaminodeisopropyl-dehydroabietate methyl ester (**1**), was prepared by sequential esterification, bromation, dinitration and reduction according to literature procedures [13]. Compound **2**, a white powder which has low solubility in methanol and is readily soluble in chloroform, was prepared in good yields (74.4%) by coupling/ oxidation of **1** with salicylaldehyde in dioxane using air as the oxidant. The salient features of the coupling/oxidation include a simple procedure, mild conditions, no need for a commercial oxidant/ additive, no waste produced (the only by-product being water) and easy purification. The structure of **2** was confirmed by ^1^H-NMR, ^13^C-NMR, MS, IR and elemental analysis.

**Figure 1:**
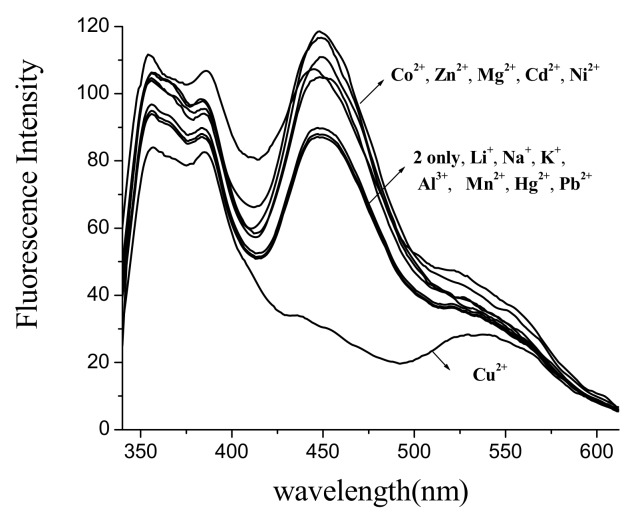
Fluorescence spectra for compound **2** excited at 350 nm in the absence and presence of different metal cations in THF. The concentrations of **2** and metal ion were both 1×10^−5^ mol/L.

**Figure 2:**
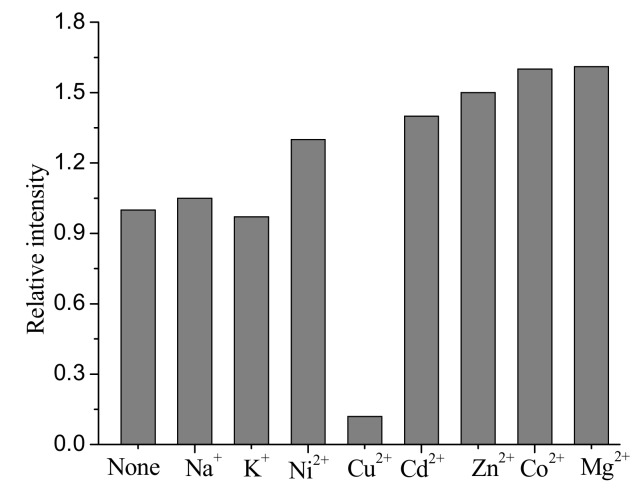
Relative fluorescence intensity at the metal ion to compound **2** mole ratio of 2.0 (almost the same even at 1.0) in the titration of **2** with different metal ions.

The influence of different metal cations on the fluorescence intensity of compound **2** was investigated in THF. As shown in Figure 1, free 2-(2′-hydroxyphenyl)benzimidazole **2** exhibits three emissions centered at 355, 384 and 448 nm. The fluorescence intensity of the 448 nm band was significantly quenched and a new peak at 540 nm was observed in the presence of Cu^2+^. A fluorescence enhancement was observed in the presence of Mg^2+^, Co^2+^, Ni^2+^, Zn^2+^ and Cd^2+^, respectively, while no significant spectral changes of **2** occurred in the presence of other cations such as Li^+^, Na^+^, K^+^, Al^3+^, Mn^2+^, Hg^2+^ and Pb^2+^. Also, based on the relative fluorescence intensities shown in Figure 2, it can be concluded that Cu^2+^ is selectively recognized by **2** among the studied metal ions.

**Figure 3:**
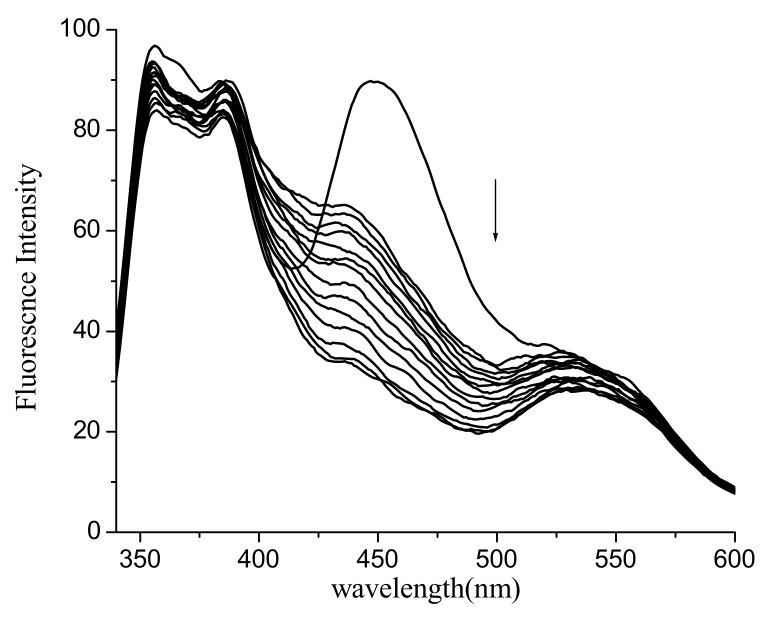
Fluorescence spectral changes of **2** (λ_ex_ = 350 nm) upon the addition of Cu^2+^ in THF at 298 K.

**Figure 4:**
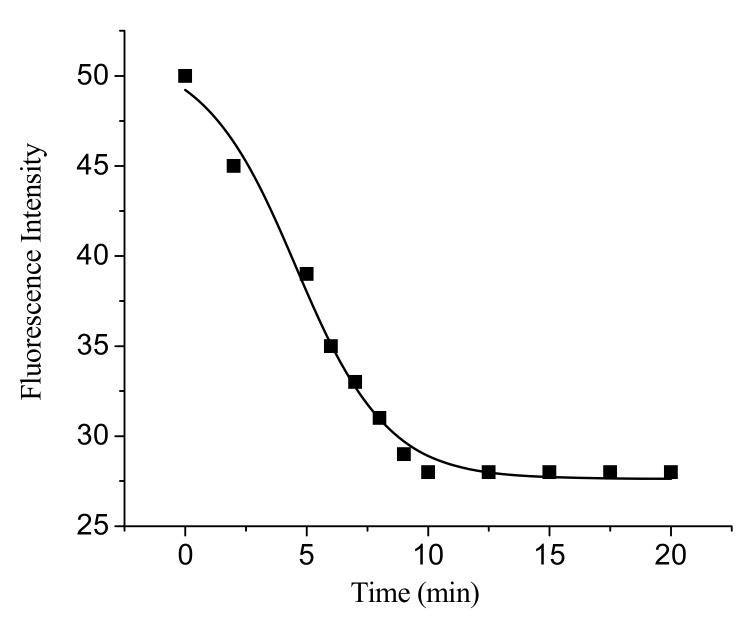
Kinetic behavior of the fluorescence of the **2**-Cu^2+^ reaction system in THF solution. [**2**] = 1.00 × 10^−5^ M, [Cu^2+^] = 1.00 × 10^−5^ M.

**Figure 5:**
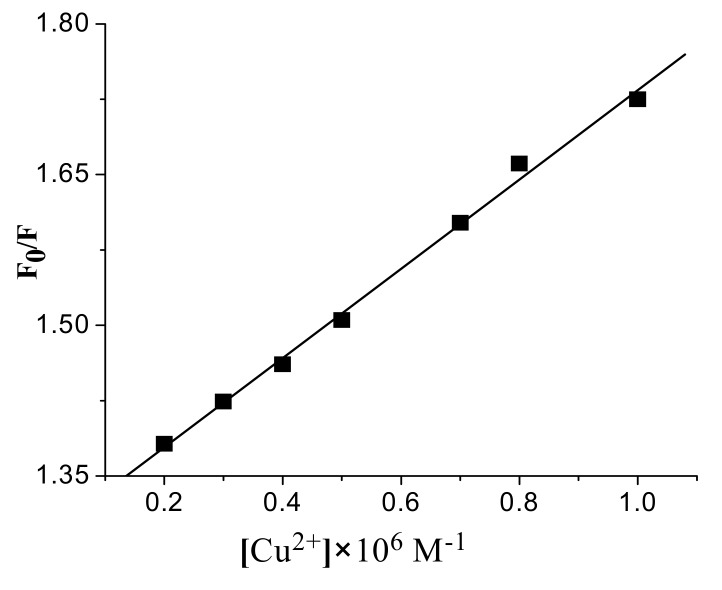
The Stern-Volmer plots of **2**-Cu^2+^ system at 298K.

The changes in fluorescence intensity of **2** upon addition of Cu^2+^ in THF solution were recorded. Figure 3 shows the fluorescence spectral changes of **2** (λ_ex_ = 350 nm) upon the addition of Cu^2+^ in THF at 298 K. As mentioned, this addition resulted in a remarkable decrease of the fluorescence signal corresponding to the benzimidazole moiety at 448 nm, and the appearance of a new emission peak at 540 nm attributed to the formation of a benzimidazole-copper compound. Figure 4 shows the kinetic characteristics of the reaction in THF solution. When the reaction time of Cu^2+^ (1.00 × 10^−5^ M) with **2** (1.00 × 10^−5^ M) in THF was longer than 10 min, a stable fluorescence signal was observed and it remained constant for at least 12 h. Higher concentrations of either Cu^2+^ or **2** will be of benefit to shorter reaction times. These results prove the formation of a complex of **2** with Cu^2+^. The fluorescence quenching data are analyzed by the Stern-Volmer equation [14]:

F_0_ / F = 1 + K_sv_*c*(Q) (1)

where F_0_ and F denote the fluorescence intensities before and after the addition of the quencher, respectively, *c*(Q) denotes the concentration of quencher, and K*sv* is the Stern-Volmer quenching constant. Figure 5 displays the Stern-Volmer plots of **2**-Cu^2+^ system at 298 K. The plots showed that within the investigated concentrations, the results agreed with the Stern-Volmer Eq. (1). And the quenching constant K*sv* of **2** to Cu^2+^ obtained from the Stern-Volmer equation is 4.44 × 10^5^ L mol^−1^.

**Figure 6:**
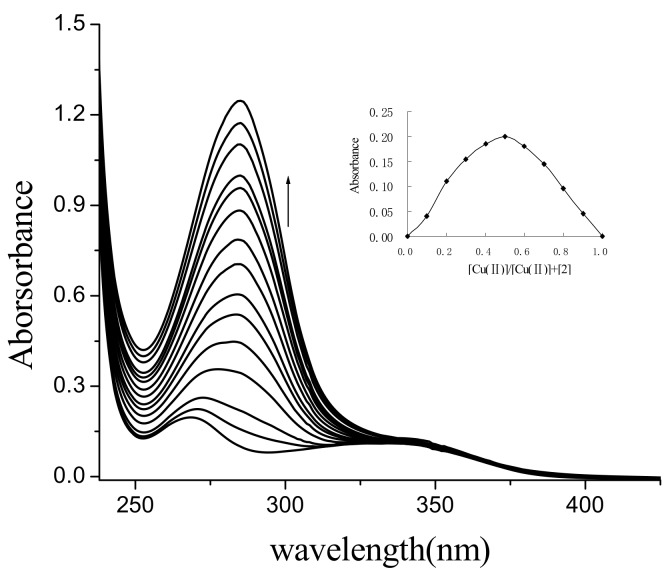
UV-Vis spectral changes of **2** upon the addition of Cu^2+^ ion. [**2**] = 1.00 × 10^−5^ M, [Cu^2+^] = 0~2 × 10^−5^ M. Inset shows the job’s plots for complexation of **2** with Cu^2+^ determined by UV-Vis spectroscopy in THF. [**2**] + [Cu^2+^] = 1.0 × 10^−5^ M at 298 K.

The UV-Vis absorption spectra of **2** upon addition of Cu^2+^ is shown in Figure 6. Two absorption peaks of **2** were observed at 267 and 337 nm with molar extinction coefficients *ε* values (10^4^ M^−1^ cm^−1^) in agreement with π-π* transitions. Upon gradual addition of increasing concentrations of Cu^2+^ (0~2 equiv.) to the solution of **2**, the absorption peak at 276 nm was dramatically increased and shifted to longer wavelength (17 nm), whereas no significant change was observed at the 338 nm absorption peak. The stoichiometry of the complex between **2** and Cu^2+^ was established by Job’s plots as shown in Figure 6 (inset). The maximum for complexation of **2** with Cu^2+^ at a mole fraction of 0.5 indicates 1:1 complex formation.

## 3. Experimental

### 3.1. General

Fluorescence spectra measurements were acquired on a RF-5301PC spectrophotometer, equipped with a xenon discharge lamp and a 1 cm quartz cell. The UV-Vis absorption spectra were acquired using a CARY 100 spectrophotometer. NMR spectra were recorded in CDCl_3_ on a Bruker AVANCE-500 MHz NMR spectrometer with TMS as internal standard. Elemental analyses were performed on a Carlo Erba model 1106 elemental analyzer. IR spectra were acquired on IR spectra were recorded on a Nicolet ESP 360 FT-IR instrument. Mass spectra were obtained on a Bruker Esquire HCT spectrometer. All of the chemicals used in this work were of analytical grade purchased from commercial suppliers and used without further purification. The solutions of the metal ions were prepared from their corresponding nitrate or chloride salts. The stock solution of compound **2** (1.00 × 10^−4^ M) was prepared in a 100 mL volumetric flask, by dissolving **2** (4.83 mg) in THF, and diluting to the mark with THF, and the required concentration then being obtained by accurate dilution. The metal ions stock solutions (1.00 × 10^−2^ M) were prepared in THF. 

### 3.2. Preparation of Methyl 2′-hydroxyphenyl-12-bromo-13,14-imidazolyl-deisopropyldehydroabietate (**2**).

12-Bromo-13,14-diaminodeisopropyldehydroabietate methyl ester (190 mg, 0.5 mmol) and salicylaldehyde (61 mg, 0.5 mmol) were dissolved in dioxane (15 mL). After refluxing for 5 h, a white precipitate was formed. The solid was filtered and purified with re-crystallization from methanol to give white needle-shaped crystals of **2** (0.18 g, 74.4%), m.p. 172-172.8 °C; ^1^H-NMR (CDCl_3_) δ: 1.29 (s, 3H), 1.34 (s, 3H), 1.52-1.59 (m, 2H), 1.71-1.81 (m, 5H), 2.23-2.32 (m, 2H), 2.74-2.75 (m, 2H), 3.03 (m, 1H), 3.70 (s, 3H), 6.94 (s, 1H), 7.00-7.02 (m, 1H), 7.07 (d, *J* = 8.55Hz, 1H), 7.44-7.47 (m, 2H), 8.45 (s, 1H), 12.71 (s, 1H ); ^13^C-NMR (CDCl_3_) δ: 16.6, 18.5, 24.5, 25.2, 29.3, 36.6, 37.8, 38.2, 44.1, 47.6, 52.3, 117.5, 119.2, 119.5, 124.1, 128.8, 126.9, 132.8, 134.0, 147.7, 150.4, 152.1, 161.0, 164.0, 178.7; IR (KBr) υ_max_: 2936, 1724, 1635, 1361, 1248, 1114, 742 cm^−1^; Anal. Calcd. for C_25_H_27_BrN_2_O_3_, C 62.12, H 5.63, N 5.80; found: C 62.24, H 5.58, N 5.75; positive ACPIMS m/z (relative intensity) 484.2 [M + H]^+^ (100).

## 4. Conclusions

A dehydroabietyl derivative **2** bearing a 2-(2′-hydroxyphenyl)benzimidazole unit was synthesized using methyl dehydroabietate as a starting material via several steps involving bromation, dinitration, reduction and coupling/oxidation. The structure of compound **2** was characterized by ^1^H-NMR, ^13^C-NMR, IR, MS and elemental analysis. Its sensing behaviors toward metal ions (Li^+^, Na^+^, K^+^, Mg^2+^, Co^2+^, Ni^2+^, Zn^2+^, Cu^2+^, Cd^2+^, Al^3+^, Mn^2+^, Hg^2+^ and Pb^2+^) were investigated by a fluorescence spectroscopy method, and the fluorescence intensity was only quenched by Cu^2+^. The results show that compound **2** may be applicable as a fluorescent chemosensor for Cu^2+^.

## Supplementary Materials

Supplementary Materials can be found at http://www.mdpi.com/1420-3049/16/1/100/s1.
