# Supplementary material for: Synthesis of a Dehydroabietyl Derivative Bearing a 2-(2′-Hydroxyphenyl)Benzimidazole Unit and Its Selective Cu2+ Chemosensing
Source: Molecules. 2010 Dec 28;16(1):100–6. doi: 10.3390/molecules16010100 (PMC6259128; doi:10.3390/molecules16010100)
Supplement: Supplementary file 1 [file molecules-16-00100-s001.pdf]

(1) The ^{1}H NMR spectra of compound 2

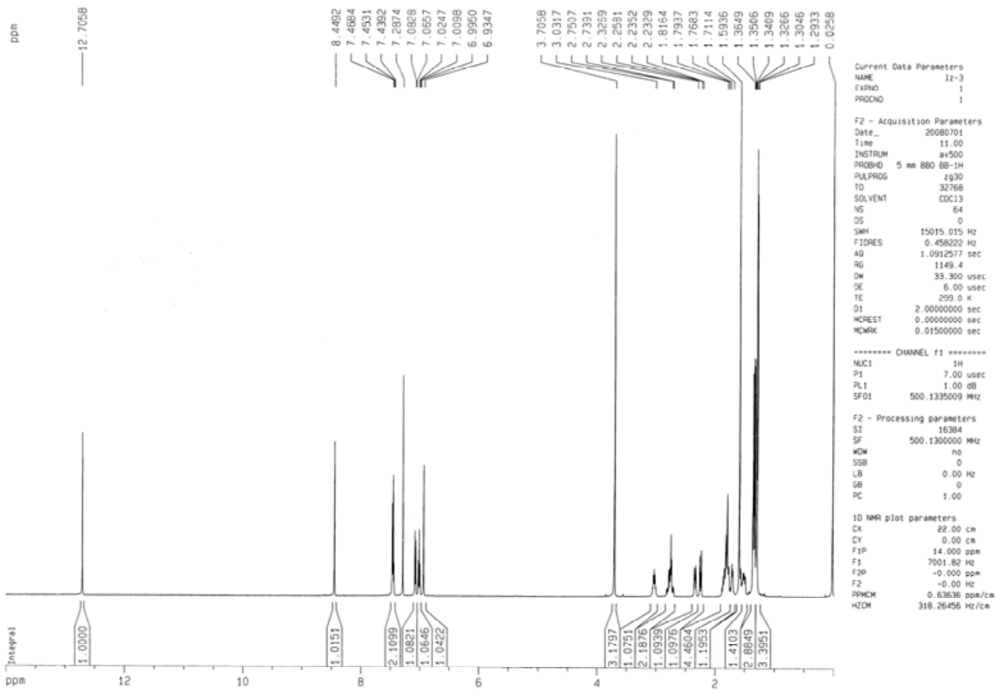

(2) The ^{13}C NMR spectra of compound 2

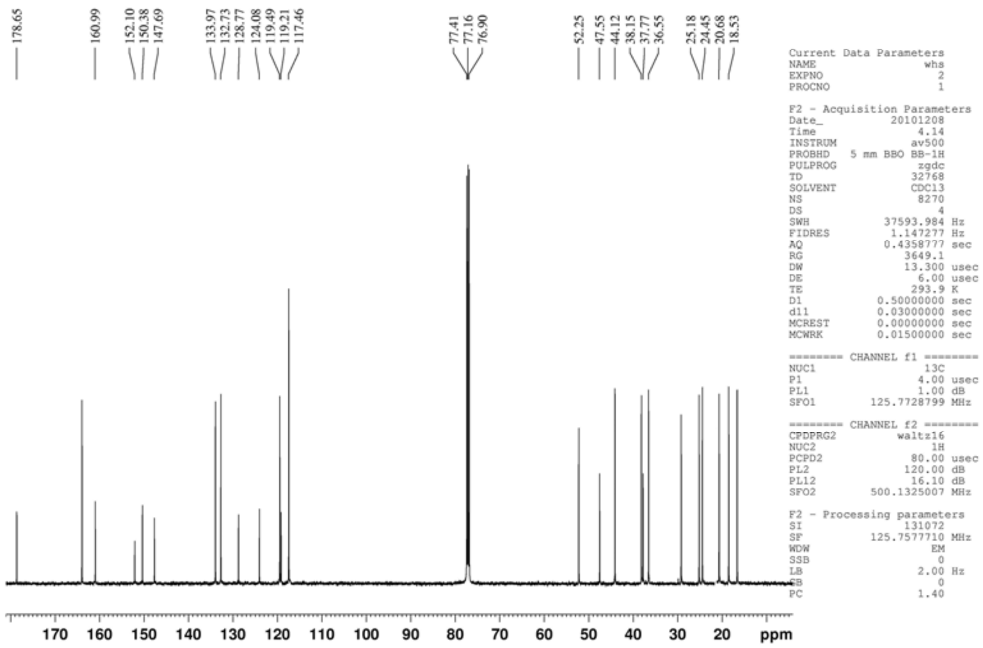

(3) The Mass spectra of compound 2

Mass Spectrum List Report

Analysis Info

Analysis Name	D:\Data\wanghengshan\101207\101207-482-01.d		
Method	APCI_POS_LOW.m	Operator	hct
Sample Name	Dummy	Instrument	HCT
Comment			

Acquisition Parameter

Ion Source Type	APCI	Ion Polarity	Positive	Alternating Ion Polarity	off
Mass Range Mode	Std/Enhanced	Scan Begin	50 m/z	Scan End	1000 m/z
Capillary Exit	166.0 Volt	Skimmer	40.0 Volt	Trap Drive	90.7
Accumulation Time	45 µs	Averages	8 Spectra	Auto MS/MS	off

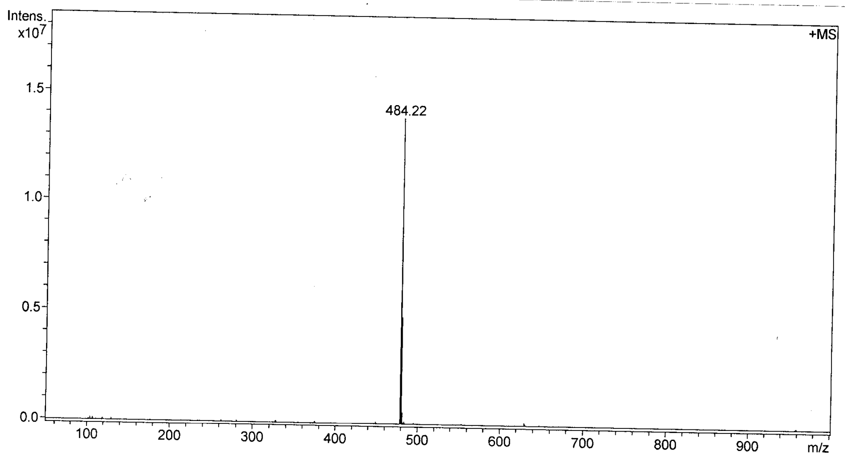

#	m/z	I	I %
1	104.62	104592	0.8
2	107.72	113057	0.8
3	119.75	75863	0.5
4	449.21	87463	0.6
5	484.22	13896395	100.0
6	485.23	4826552	34.7
7	486.16	532974	3.8
8	486.32	418894	3.0
9	488.95	118851	0.9
10	633.33	138180	1.0
